# Effectiveness of overuse injury prevention programs on upper extremity performance in overhead youth athletes: A systematic review

**DOI:** 10.1016/j.smhs.2023.03.001

**Published:** 2023-03-17

**Authors:** Rachel Y. Lau, Swarup Mukherjee

**Affiliations:** Physical Education and Sport Science Academic Group, National Institute of Education, Nanyang Technological University Singapore, 1 Nanyang Walk, 637616, Singapore

**Keywords:** Youth sports, Overhead athlete, Overuse injury, Injury prevention, Shoulder, Elbow

## Abstract

Several targeted upper extremity injury prevention programs have been developed to mitigate the risk of upper extremity overuse injuries among youth athletes in overhead sports; however, their effectiveness on performance outcome measures has not been investigated. This systematic review evaluated the effectiveness of existing upper extremity injury prevention programs that focused on modifying intrinsic risk factors, and performance outcome measures in overhead youth athletes. The secondary aim was to identify the training components of these programs. PubMed, Physiotherapy Evidence Database (PEDro), SPORTDiscus (via EBSCOhost), and Web of Science were searched from January 2000 to November 2020 for studies that implemented training programs or exercises for upper extremity injury prevention among youth athletes in overhead throwing or striking sports. An updated search was conducted from December 2020 to October 2022. A program was deemed effective for a performance outcome measure if significant improvements were observed in the intervention group as compared to the control group. Of the 1 394 studies identified, five studies met the inclusion criteria. The effectiveness of the injury prevention programs on the identified performance outcome measures of strength, mobility, and sport-specific measures were 30.4%, 28.6%, and 22.2%, respectively. The training components targeted were strength, mobility, and plyometrics. Strength was the most common training component and was also the most widely investigated performance outcome measure. Overall, current upper extremity injury prevention programs seem effective at improving performance outcome measures of strength, mobility, and sport-specific outcomes with training components of strength, mobility and plyometrics. Standardized protocols are required for the measurement and reporting of performance outcomes measures, and the reporting of training components.

## Abbreviation

D: dominant
ER: external rotation
GIRD: glenohumeral internal rotation deficit
HHD: hand-held dynamometer
HA: horizontal adduction
IPP: injury prevention program
IR: internal rotation
mYKB-9: modified Yokohama Basbeall-9
ND: non-dominant
PEDro: Physiotherapy Evidence Database
PRISMA: Preferred Reporting Items for Systematic Reviews and Meta-Analyses
RCT: randomized controlled trial
RIO: High School Reporting Information Online
ROM: range of motion
YKB-9: Yokohama Baseball-9

## Introduction

Upper extremity overuse injuries are a growing concern for all athletes in overhead throwing or striking sports.^1^ This stems mainly from the nature of overhead sports, where the hand is repeatedly raised above the head to perform a forceful throwing or striking action.^2^^,^^3^ However, for youth athletes (9- to 18-years old) characterised by musculoskeletal immaturity, this repetitive act renders them even more susceptible to overuse injuries compared to their adult counterparts.^4^^,^^5^ The risk of sustaining overuse injuries in youth athletes is further magnified due to practices like early sport specialization and developmentally inappropriate training load in youth sports.^6^^,^^7^

A high prevalence of upper extremity (i.e., shoulder and elbow) overuse injuries has been reported in overhead youth sports. A descriptive epidemiological study on 15- to 18-years old female volleyball athletes observed a 40% prevalence of shoulder pain that was not associated with a traumatic injury.^8^ Another 34-week prospective cohort study on elite 16- to 18-years old male handball players found the average prevalence of shoulder overuse injuries to be 17% (95% *CI* [16%–19%]), with a relative burden of 33% (summed severity score of the shoulder as a proportion of the total severity score of all overuse injuries recorded).^9^ Collectively, these studies reflect a high burden magnitude of upper extremity overuse injuries among competitive overhead youth athletes. With pain, physical discomfort, movement limitations, reduced participation ability, reduced performance, and growth disturbances identified as consequences of upper extremity overuse injuries,^10^^,^^11^ there is a clear need for the stakeholders to address these injuries, and their prevention, as a priority.

Based on the popular ‘sequence of prevention’,^12^ the development of an injury prevention program (IPP) should be informed by previously identified risk factors of the injury. Consequently, any reduction in injury risk should be a result of mitigating the identified risk factors, which can be observed via improvements in performance outcome measures such as strength and mobility.^13^ In this regard, systematic reviews have evaluated existing IPPs focusing on modifiable intrinsic risk factors through training programs and/or exercises, and their resultant improvements in performance outcome measures among youth athletes. However, the majority of the reviews have focused on lower-extremity IPPs.^14^^,^^15^ Apparently, no systematic review currently exists that has investigated the effectiveness of these types of upper extremity IPPs on performance outcome measures in the vulnerable population of overhead youth athletes. Identifying the components of effective IPPs would also be purposeful to enhance our understanding and improve the development of future IPPs.^14^^,^^16^

The aim of this systematic review was to investigate the effectiveness of upper extremity IPPs that focus on modifying intrinsic risk factors, and on performance outcome measures in overhead youth athletes. A secondary aim was to identify the training components targeted by the existing upper extremity IPPs. The findings from this systematic review can provide greater insights into the planning and development of future IPPs and determine their effectiveness on risk mitigation and sports performance in overhead youth athletes.

## Methods

### Search strategy

An electronic search was conducted according to the guidelines of the Preferred Reporting Items for Systematic Reviews and Meta-Analyses (PRISMA).^17^ PubMed, Physiotherapy Evidence Database (PEDro), SPORTDiscus (via EBSCOhost), and Web of Science were searched from January 2000 to November 2020 for relevant studies. Thereafter, an updated search was conducted from December 2020 to October 2022. Keywords used included, youth, children, junior, young, adolescent, injury, athletic injury, sports injury, overuse injury, prevention, prevention program, prevention exercises, prevention training, upper extremity, arm, shoulder, elbow, outcomes, outcome measures, performance, performance measures, performance outcomes. The detailed search strategy is illustrated in Table 1. To ensure contextual relevance, database filters applied included: published in the English language, published in peer-reviewed academic journals, and published from Year 2000 onward. The reference lists of included studies were manually checked for any relevant studies that were not identified during initial database search.

**Table 1:** Search strategy.

Database		Search string
PubMed	1	Youth athletic upper extremity injury prevention outcomes
	2	(Youth OR junior OR adolescent OR children) AND (shoulder OR elbow OR arm) AND ((athletic OR sports) injury) AND (prevention (training OR program∗ OR exercise∗)) AND outcomes
	3	(Youth OR junior OR adolescent OR children) AND (shoulder OR elbow OR arm) AND ((athletic OR sports) injury) AND (prevention (training OR program∗ OR exercise∗)) AND performance outcomes
	4	(Youth OR junior OR adolescent OR children) AND (shoulder OR elbow OR arm) AND ((athletic OR sports) injury) AND (prevention (training OR program∗ OR exercise∗)) AND performance measures
	5	(Youth OR junior OR adolescent OR children) AND (shoulder OR elbow OR arm) AND ((athletic OR sports) injury) AND (prevention (training OR program∗ OR exercise∗)) AND outcome measures
	6	(Youth OR junior OR adolescent OR children) AND (shoulder OR elbow OR arm) AND ((athletic OR sports) injury) AND (prevention (training OR program∗ OR exercise∗)) AND performance
PEDro	1	Youth injury prevention
	2	Junior injury prevention
	3	Youth injury performance measures
	4	Youth injury performance outcomes
	5	Youth injury outcome measures
	6	Youth athletic injury
	7	youth sport injury prevention
SPORTDiscus via EBSCOhost	1	Youth AND ((athletic OR sports) injury) AND prevention AND (outcome measures)
	2	Youth AND ((athletic OR sports) injury) AND prevention AND (performance measures)
	3	Youth AND ((athletic OR sports) injury) AND prevention AND (performance outcomes)
	4	(Youth OR junior OR adolescent OR children) AND (shoulder OR elbow OR arm) AND ((athletic OR sports) injury) AND (prevention (training OR program∗ OR exercise∗))
	5	(Youth OR junior OR adolescent OR children) AND (shoulder OR elbow OR arm) AND ((athletic OR sports) injury) AND prevention
Web of science	Set 1	ts= (outcomes OR “outcome measures” OR performance OR “performance measures” OR “performance outcomes”)
	AND Set 2	ts=(prevention OR training OR program∗ OR exercise∗ OR “prevention training” OR “prevention program∗” OR “prevention exercise∗”)
	AND Set3	ts=(injury OR “athletic injuries” OR “sports injuries”)
	AND Set 4	ts=(shoulder OR elbow OR arm OR upper OR extremit∗ OR “upper extremit∗”)
	AND Set 5	ts=(Youth OR junior OR adolescent OR children)

### Study selection

Duplicates of studies identified from the search strategy were removed. Titles and abstracts of remaining studies were screened to determine eligibility. The inclusion criteria were based on the Population, Intervention, Comparison, Outcome, Study design (PICOS) concept and are as follows: participants were youth athletes (9- to 18-years old) with full participation in forceful overhead throwing or striking sports, the intervention utilized training programs or exercises for the primary prevention of upper extremity injury with a control group performing usual training or sham exercises, at least one performance outcome measure was assessed (e.g., strength, mobility), and the studies utilized randomized controlled trials (RCTs), cluster-RCT, or non-randomized controlled trials (non-RCTs). Studies were excluded if participants were 8 years and below or 19 year old and above, the sample included participants who could not fully participate in normal training session and results could not be separated, interventions were passive in nature (i.e., equipment or legislative changes), training programs or exercises were focused on reinjury prevention or only focused on performance enhancement without consideration for injury prevention. Where titles and abstracts of studies were insufficient to confirm eligibility, they were included in the full-text evaluation. RL and SM evaluated the studies against the inclusion criteria and disagreements were resolved by discussion.

### Data extraction and synthesis

Data extracted from all eligible studies by the authors RL and SM included the study design, the number of participants, participants’ demographics, details of the IPP, performance outcome measures used, and effectiveness of the IPP on the performance outcome measures. Any discrepancies were resolved through discussion.

The effectiveness of a program in mitigating intrinsic risk factors was determined by significant improvements in the respective performance outcome measure(s) in the intervention group as compared to the control group.^18^ Performance outcome measures were also classified into categories to further understand the measures of interest to researchers. Where data was not reported in the study, corresponding authors were contacted via email.

Based on the details of exercises included in each program, training components were identified to obtain further insights into existing IPPs. As no previous work seemingly exists on the categorisation of training components for upper extremity IPPs, the categories used in this review were adapted from previous work on soccer IPPs.^19^ Only three of the six categories of training components, strength, mobility and plyometrics, were relevant and suitable for use for upper extremity IPPs.

### Methodological quality

The included studies were assessed for quality independently by the authors (RL and SM) using the PEDro Scale.^20^ This tool is of an appropriate construct to evaluate the quality of RCTs^21^ and generates acceptable inter-rater reliability.^22^ The PEDro Scale is a checklist of Yes/No questions used to examine particular aspects of research methodology, including key aspects of internal validity. If the statements matched the evaluated study, a ‘‘yes’’ answer added 1 point, and if it did not, a ‘‘no’’ answer added 0 points. The external validity of the articles is represented by Item-1 of the PEDro Scale, as “eligibility criteria were specified”, and is excluded in the tabulation of the final score. Therefore, although there are 11 items on the scale, the maximum score possible on the PEDro scale is 10 (with the exclusion of Item-1), and a score of ≥ 6 reflects adequate methodological quality.^23^

## Results

**Fig. 1:**
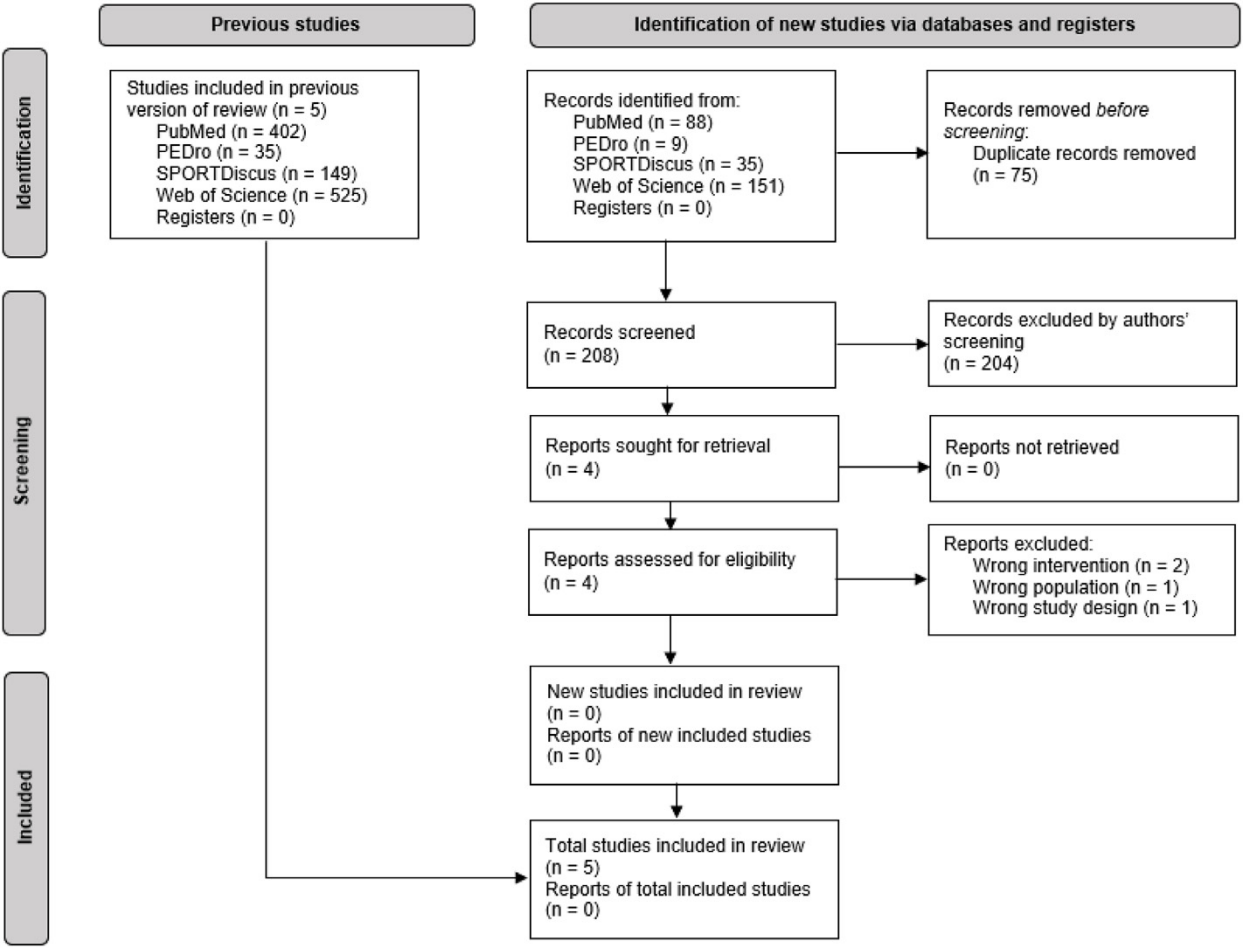
Study identification, screening, and exclusion pathway.

The initial database search identified 1 111 studies. After removing duplicates and excluding irrelevant studies based on titles and abstracts, 17 studies were identified for full-text review. Twelve studies were subsequently excluded as they did not meet the eligibility criteria. The reference lists of the remaining five studies were screened for potentially suitable studies. One additional study was identified and a total of six studies were considered eligible for this review. However, despite repeated attempts to contact the corresponding author, the data for one study could not be obtained.^24^ Therefore, the study was subsequently dropped from inclusion and ultimately a total of five studies were considered for analysis.^25^, ^26^, ^27^, ^28^, ^29^ The updated search identified 283 studies, of which 75 were duplicates. Following titles and abstracts screening, four studies were included for full-text review, of which none met the eligibility criteria. Fig. 1 shows the flowchart for study selection.

### Study characteristics

A summary of the included studies is presented in Table 2. All studies utilized an RCT design. Sports included were handball, baseball, tennis, and cricket. The number of players in the included studies ranged from 25 to 219, with a total sample size of 378 for this review. Overall, the participants included more males (*n* ​= ​296) than females (*n* ​= ​82). The reported mean age of participants ranged from 10.2 to 15.9 years.

**Table 2:** Characteristics of included studies.

Author	Study design, Sport	Participants (*n*_IG_; *n*_CG_)	Age (years)	Upper extremity performance outcome measures	Results			
					IG		CG	
					Pre	Post	Pre	Post
Fernandez-Fernandez et al.^28^	RCT, Tennis	30 males (15; 15)	IG: 13.2 ​± ​0.6	Serve velocity [km/h]	150.3 ​± ​12.3	157.9 ​± ​12.5^a^	146.1 ​± ​10.7	146.6 ​± ​10.4
			CG: 13.2 ​± ​0.5					
			Range: around 13					
				Shoulder total ROM [°]	166.0 ​± ​20.2	179.6 ​± ​14.0^b^	161.4 ​± ​15.5	169.6 ​± ​14.1^b^
				Serve accuracy [points]	12.2 ​± ​2.5	13.5 ​± ​3.6	12.5 ​± ​2.5	13.5 ​± ​2.7
Forrest et al.^25^	RCT, Cricket	65 males (32; 33)	15.6 ​± ​1.1	Shoulder isokinetic eccentric ER				
			Range: 14-17					
				D at 90°/s [Nm/kg]	0.52 ​± ​0.10	0.55 ​± ​0.09^a^	0.54 ​± ​0.10	0.52 ​± ​0.09
				D at 180°/s [Nm/kg]	0.54 ​± ​0.10	0.55 ​± ​0.09	0.56 ​± ​0.08	0.53 ​± ​0.09
Mascarin et al.^26^	RCT, Handball	25 females (13, 8 D, 5 ND; 12, 7 D, 5 ND)	IG (D): 15.3 ​± ​0.9	Shoulder isokinetic concentric IR				
			IG (ND): 15.2 ​± ​0.5					
			CG (D): 15.9 ​± ​1.2					
			CG (ND): 15.4 ​± ​0.9					
			Range: not stated					
				D PT at 60°/s [Nm]	29.4 ​± ​1.0	30.6 ​± ​1.8	32.0 ​± ​1.3	26.5 ​± ​1.2^b^
				ND PT at 60°/s [Nm]	29.4 ​± ​1.0	29.1 ​± ​1.8^a^	26.8 ​± ​1.0	23.8 ​± ​0.9
				Shoulder isokinetic concentric ER				
				D PT at 60°/s [Nm]	18.0 ​± ​0.8	21.3 ​± ​1.0^b^	21.2 ​± ​1.4	22.9 ​± ​1.8
				ND PT at 60°/s [Nm]	18.0 ​± ​0.8	21.1 ​± ​1.3^b^^a^	15.6 ​± ​0.7	16.1 ​± ​1.1
				D PT at 240°/s [Nm]	16.4 ​± ​1.21	15.4 ​± ​1.1	17.8 ​± ​1.7	15.6 ​± ​2.9
				ND PT at 240°/s [Nm]	18.5 ​± ​0.8	17.5 ​± ​1.9	18.6 ​± ​1.9	17.7 ​± ​0.8
				D TW at 60°/s [J]	29.3 ​± ​0.9	34.5 ​± ​1.5^b^	34.8 ​± ​2.5	37.6 ​± ​3.1^b^
				ND TW at 60°/s [J]	29.0 ​± ​1.4	34.6 ​± ​1.6^b^^a^	25.8 ​± ​1.3	24.7 ​± ​1.6
				Shoulder isokinetic eccentric ER				
				D PT at 240°/s [Nm]	30.8 ​± ​1.2	30.2 ​± ​1.8	31.5 ​± ​1.7	32.5 ​± ​1.8
				ND PT at 240°/s [Nm]	27.6 ​± ​3.5	36.0 ​± ​1.9^b^^a^	29.9 ​± ​3.3	29.2 ​± ​1.0
				Shoulder conventional strength balance ratio at 60°/s				
				D (ERconc/IRconc)	61.2 ​± ​1.3	70.4 ​± ​3.7^a^	66.1 ​± ​20.6	86.6 ​± ​6.6^b^
				ND (ERconc/IRconc)	61.5 ​± ​3.5	72.7 ​± ​3.0^b^	58.1 ​± ​0.6	67.6 ​± ​3.7
				Shoulder functional strength balance ratio at 240°/s				
				D (ERecc/IRconc)	1.2 ​± ​0.06	1.2 ​± ​0.07	1.3 ​± ​0.06	1.4 ​± ​0.1
				ND (ERecc/IRconc)	1.0 ​± ​0.1	1.6 ​± ​0.08	1.5 ​± ​0.1	1.3 ​± ​0.05
				Ball throwing velocity				
				D standing throw [km/h]	49.0 ​± ​2.4	52.5 ​± ​2.2^b^	53.3 ​± ​1.8	52.6 ​± ​1.6
				ND standing throw [km/h]	38.1 ​± ​2.5	37.2 ​± ​1.1	36.6 ​± ​1.0	40.4 ​± ​1.2^b^
				D jumping throw [km/]	No changes between pre/post		No changes between pre/post	
				ND jumping throw [km/h]	No changes between pre/post		No changes between pre/post	
Mascarin et al.^27^	RCT, Handball	39 females (21; 18)	EG: 15.3 ​± ​1.1	Shoulder isokinetic concentric IR				
			CG: 15.0 ​± ​0.8					
			Range: not stated					
				D PT at 60°/s [Nm]	25.2 ​± ​1.1	27.1 ​± ​1.1	22.7 ​± ​1.1	24.8 ​± ​1.1
				D PT at 240°/s [Nm]	21.3 ​± ​1.0	21.6 ​± ​0.9	19.5 ​± ​1.0	19.6 ​± ​0.9
				Average power at 240°/s [W]	27.4 ​± ​2.1	30.1 ​± ​2.0^b^^a^	24.6 ​± ​2.1	23.5 ​± ​2.3
				Shoulder isokinetic concentric ER				
				D PT at 60°/s [Nm]	20.6 ​± ​0.8	22.0 ​± ​0.9	17.3 ​± ​0.8	20.0 ​± ​0.9
				Shoulder isokinetic eccentric ER				
				D PT at 240°/s [Nm]	37.6 ​± ​1.6	37.8 ​± ​1.0	32.2 ​± ​1.6	34.9 ​± ​1.0
				Shoulder conventional strength balance ratio at 60°/s				
				D (ERconc/IRconc)	82.8 ​± ​2.3	81.7 ​± ​3.0	77.6 ​± ​2.4	81.7 ​± ​3.1
				Shoulder functional strength balance ratio at 240°/s				
				D (ERecc/IRconc)	1.8 ​± ​0.1	1.8 ​± ​0.1	1.7 ​± ​0.1	1.8 ​± ​0.1
				Ball throwing speed				
				D standing throw [km/h]	49.3 ​± ​1.4	52.4 ​± ​1.4^b^	47.5 ​± ​1.4	49.7 ​± ​1.5
				D jumping throw [km/h]	56.2 ​± ​1.6	60.6 ​± ​1.4^b^	56.0 ​± ​1.6	58.5 ​± ​1.5
Sakata et al.^29^	RCT, Baseball	201 males, 18 females (109; 110)	10.2 ​± ​0.8	Ball speed [km/h]	64.3 ​± ​10.5	Δ 6.4 ​± ​6.1^a^	64.7 ​± ​9.8	Δ 4.1 ​± ​6.7
			Range: 9-11					
				Elbow extension deficits^c^ [°]	1.6 ​± ​3.3	Δ −1.5 ​± ​2.9	2.1 ​± ​3.4	Δ −0.3 ​± ​5.2
				Shoulder ROM deficits^c^				
				ER [°]	−3.8 ​± ​10.6	Δ 4.2 ​± ​12.6	−5.2 ​± ​11.4	Δ 5.6 ​± ​16.5
				IR [°]	15.0 ​± ​10.2	Δ −3.0 ​± ​11.4	13.0 ​± ​13.0	Δ −1.6 ​± ​10.4
				Shoulder total ROM [°]	150.9 ​± ​10.8	Δ 4.4 ​± ​12.4	152.4 ​± ​11.8	Δ 2.2 ​± ​12.6
				Shoulder HA deficits^c^ [°]	5.6 ​± ​6.2	Δ −5.6 ​± ​6.2^a^	5.1 ​± ​5.9	Δ −1.6 ​± ​6.1
				Thoracic kyphosis angle [°]	29.4 ​± ​5.8	Δ −3.3 ​± ​9.0^a^	29.2 ​± ​5.5	Δ 0.6 ​± ​6.9

ΔChange in respective performance outcome measures from pre-to post-intervention.

CG, control group; conc, concentric; D, dominant limb; ecc, eccentric; ER, external rotation; F, female; HA, horizontal adduction; IG, intervention group; IR, internal rotation; M, male; *n*_CG_, number of participants in the control group; *n*_IG_, number of participants in the intervention group; ND, non-dominant limb; PT, peak torque; RCT, randomized controlled trial; ROM, range of motion; TW, total work.

Values presented as mean ​± ​standard deviation unless otherwise stated.

a Statistically significant difference (*p* < 0.05) between intervention and control group (pre-post-test).

b Statistically significant difference (*p* < 0.05) within groups (pre-post-test).

c Deficits defined as the difference between non-dominant and dominant limbs.

The effectiveness of the respective IPPs on upper extremity performance outcome measures is illustrated in Table 2. Upper extremity performance outcome measures that significantly improved in the intervention group as compared to the control group were: serve velocity (tennis),^28^ dominant (D) shoulder isokinetic eccentric external rotation (ER) at 90°/s,^25^ non-dominant (ND) shoulder isokinetic concentric internal rotation (IR) peak torque at 60°/s,^26^ ND shoulder isokinetic concentric ER peak torque at 60°/s,^26^ and total work at 60°/s,^26^ ND shoulder isokinetic eccentric ER peak torque at 240°/s,^26^ D shoulder conventional strength balance ratio (concentric ER/concentric IR) at 60°/s,^26^ D shoulder isokinetic concentric IR average power at 240°/s,^27^ ball speed (baseball pitching),^29^ D shoulder horizontal adduction (HA) deficits,^29^ and thoracic kyphosis angle.^29^

## Categories of performance outcome measures

The breakdown of the upper extremity performance outcome measures into the categories of strength,^25^, ^26^, ^27^ mobility^28^^,^^29^ and sport-specific^26^, ^27^, ^28^, ^29^ outcome measures are presented in Table 3. There was a total of 23, 7, and 9 performance outcome measures in the strength, mobility, and sports-specific categories, respectively. The categorical effectiveness (significantly improved in the intervention group as compared to the control group) of the IPPs on their performance outcome measures was 30.4% (strength), 28.6% (mobility), and 22.2% (sports-specific) (Table 4).

**Table 3:** Upper extremity performance outcome measures categorised by performance category.

Performance category	Upper extremity performance outcome measures	Study
Strength	Shoulder isokinetic eccentric ER	
	D at 90°/s [Nm/kg]	Forrest et al.^25^^b^
	D at 180°/s [Nm/kg]	Forrest et al.^25^
	D PT at 240°/s [Nm]	Mascarin et al.^26^; Mascarin et al.^27^
	ND PT at 240°/s [Nm]	Mascarin et al.^26^^b^
	Shoulder isokinetic concentric IR	
	D PT at 60°/s [Nm]	Mascarin et al.^26^; Mascarin et al.^27^
	ND PT at 60°/s [Nm]	Mascarin et al.^26^^b^
	D PT at 240°/s [Nm]	Mascarin et al.^27^
	Average power at 240°/s [W]	Mascarin et al.^27^^b^
	Shoulder isokinetic concentric ER	
	D PT at 60°/s [Nm]	Mascarin et al.^26^; Mascarin et al.^27^
	ND PT at 60°/s [Nm]	Mascarin et al.^26^^b^
	D PT at 240°/s [Nm]	Mascarin et al.^26^
	ND PT at 240°/s [Nm]	Mascarin et al.^26^
	D TW at 60°/s [J]	Mascarin et al.^26^
	ND TW at 60°/s [J]	Mascarin et al.^26^^b^
	Shoulder conventional strength balance ratio at 60°/s	
	D (ERconc/IRconc)	Mascarin et al.^26^^b^; Mascarin et al.^27^
	ND (ERconc/IRconc)	Mascarin et al.^26^
	Shoulder functional strength balance ratio at 240°/s	
	D (ERecc/IRconc)	Mascarin et al.^26^; Mascarin et al.^27^
	ND (ERecc/IRconc)	Mascarin et al.^26^
Mobility (ROM)	Shoulder total ROM [°]	Fernandez-Fernandez et al.^28^; Sakata et al.^29^
	Elbow extension deficits^a^ [°]	Sakata et al.^29^
	Shoulder ROM deficits^a^	
	ER [°]	Sakata et al.^29^
	IR [°]	Sakata et al.^29^
	Shoulder HA deficits^a^ [°]	Sakata et al.^29^^b^
	Thoracic kyphosis angle [°]	Sakata et al.^29^^b^
Sport-specific	Serve velocity/Ball speed/Ball velocity [km/h]	Fernandez-Fernandez et al.^28^^b^; Sakata et al.^29^^b^; Mascarin et al.^26^; Mascarin et al.^27^
	Ball throwing velocity	
	ND standing throw [km/h]	Mascarin et al.^26^
	D jumping throw [km/]	Mascarin et al.^26^; Mascarin et al.^27^
	ND jumping throw [km/h]	Mascarin et al.^26^
	Serve accuracy [points]	Fernandez-Fernandez et al.^28^

conc, concentric; D, dominant limb; ND, non-dominant limb; ecc, eccentric; ER, external rotation; HA, horizontal adduction; IR, internal rotation; PT, peak torque; ROM, range of motion; TW, total work.

a Deficits defined as the difference between dominant and non-dominant limbs∖.

b Statistically significant difference (*p* < 0.05) between intervention and control group (pre-post-test).

**Table 4:** Effectiveness of each outcome measure categorised by performance category.

Performance category	No. of effectively affected performance outcome measures	No. of non-effectively affected performance outcome measures	Total	Effectiveness^a^(%)
Strength	7	16	23	30.4
Mobility	2	5	7	28.6
Sport-specific	2	7	9	22.2

a Effectiveness was determined by significant improvements (*p* < 0.05) in the intervention group as compared to the control group for the respective performance outcome measure(s).

### Details of injury prevention programs

A detailed summary of the IPPs utilized in the studies is presented in Table 5. Three studies identified their IPPs to be strength training programs.^26^, ^27^, ^28^ One study identified their program as an exercise-based IPP that was performed as an alternative to normal warm-up for training sessions,^25^ and is therefore considered as a warm-up exercise program for this review. The final study in this review utilized the modified Yokohama Baseball-9 (mYKB-9) program,^29^ which is an improved version of the original Yokohama Baseball-9 (YKB-9) program,^24^ including stretching, dynamic mobility, and lower extremity balance.

**Table 5:** Details of the injury prevention programs and the targeted training components for the upper extremity.

Study	Type of injury prevention program	Volume^a^	Equipment required	Exercises included in the program	Targeted training components		
					Strength of the upper extremity	Mobility (ROM) of the upper extremity	Plyometric of the upper extremity
Fernandez-Fernandez et al.^28^	Strength training program	3 ​× ​per week, (60–70 ​min); 6 weeks	Elastic tubing and medicine ball	Core: crunches, reverse crunches, oblique crunches, plank, side plank	Included	Not included	Included
				Elastic tubing: triceps (elbow extension), rowing, external rotation with shoulder flexed 90°, external rotation with shoulder abducted 90°, shoulder abduction to 90°, diagonal pattern flexion, reverse throw, standard forward throw, wrist flexion-extension			
				Medicine ball: chest pass, overhead throw, ear throw, squat to thrust, overhead slam, diagonal wood-chop, close-stance throw			
Forrest et al.^25^	Warm-up exercise program	2 ​× ​per week (10–15 ​min); 8 weeks	Cricket balls and resistance bands	Dynamic warm up, shoulder external rotation strengthening, hip adductor strengthening, Nordic hamstring strengthening, single-leg ball throw, squats, lunges, prone-hold	Included	Not included	Not included
Mascarin et al.^26^	Strength training program	3 ​× ​per week; 6 weeks	Resistance bands	Shoulder external rotation strengthening in (1) standing position with 90° shoulder abduction and 90° elbow flexion and (2) standing position with shoulder in neutral position and elbow flexed at 90°	Included	Not included	Not included
Mascarin et al.^27^	Strength training program	3 ​× ​per week; 6 weeks	Resistance bands	Shoulder internal rotation strengthening in (2) standing position with 90° shoulder abduction and 90° elbow flexion and (2) standing position with shoulder in neutral position and elbow flexed at 90°	Included	Not included	Not included
Sakata et al.^29^	Modified Yokohama Baseball-9 (mYKB-9)	At least 1 ​× ​per week (10 ​min); 12 months	None	Stretching: massage of brachial muscles, stretch of pronator muscles, posterior shoulder stretch, anterior shoulder stretch, posterior hip stretch	Not included	Included	Not included
				Dynamic mobility: cat and dog exercise, trunk rotation exercise			
				Lower extremity balance: lateral slide exercise, elbow-to-knee exercise			

mYKB-9, modified Yokohama Baseball-9.

a Frequency (duration of program during training, if applicable), length of study.

### Training components

Overall, three training components were targeted by the five IPPs – strength, mobility, and plyometrics. In some instances, an exercise was categorised under several training components. For instance, the ‘medicine ball overhead slam’ exercise performed in the program by Fernandez-Fernandez et al.^28^ targets both strength and plyometric components. While four IPPs targeted strength as the training component,^25^, ^26^, ^27^, ^28^ mobility was targeted as a training component in only one IPP.^29^ Similarly, plyometrics was targeted in only one IPP.^28^ Detailed information about the exercises and targeted training components of each IPP for the upper extremity is presented in Table 5.

### Methodological quality

The methodological quality of the five included studies is presented in Table 6. The average score on PEDro scale was 6.8 (adequate methodological quality ≥ 6), with a highest and lowest score of 8^29^ and 6,^25^, ^28^ respectively. The three common methodological deficits identified by the PEDro scale were the failure to blind all subjects, all therapists (who administered the therapy), and all assessors (who measured at least one key outcome).

**Table 6:** Methodological quality of included studies using the PEDro scale.

Item No.	Item	Fernandez-Fernandez et al.^28^	Forrest et al.^25^	Mascarin et al.^26^	Mascarin et al.^27^	Sakata et al.^29^
1	Eligibility criteria were specified^a^	1	1	1	1	1
2	Subjects were randomly allocated to groups	1	1	1	1	1
3	Allocation was concealed	0	1	1	1	1
4	The groups were similar at baseline regarding the most important prognostic indicators	1	0	1	1	1
5	There was blinding of all subjects	0	0	0	0	0
6	There was blinding of all therapists who administered the therapy	0	0	0	0	0
7	There was blinding of all assessors who measured at least one key outcome	0	0	0	0	1
8	Measures of at least one key outcome were obtained from more than 85% of the subjects initially allocated to groups	1	1	1	1	1
9	All subjects for whom outcome measures were available received the treatment or control condition as allocated or, where this was not the case, data for at least one key outcome was analyzed by “intention to treat”	1	1	1	1	1
10	The results of between-group statistical comparisons are reported for at least one key outcome	1	1	1	1	1
11	The study provides both point measures and measures of variability for at least one key outcome	1	1	1	1	1
	Total PEDro score	6	6	7	7	8

a This item is not included in the calculation of the PEDro score. The PEDro score includes Items 2 to 11.

## Discussion

This systematic review evaluated the effectiveness of existing upper extremity IPPs in modifying performance outcome measures in overhead youth athletes. The various performance outcome measures identified in the studies were classified into three categories – strength, mobility, and sport-specific performance measures. Performance outcome measures of strength had the highest effectiveness rate (30.4%) followed by mobility-based (28.6%) and sport-specific outcomes (22.2%) These rates suggest an apparent beneficial effect of upper extremity IPPs in improving performance outcome measures.^18^

The secondary aim was to identify the training components targeted by the existing IPPs. The three training components identified were strength, mobility, and plyometrics. Strength was the most popular targeted training component across the studies (four out of five studies), consistent with its associated performance outcome measures being the most widely investigated.

### Strength-based performance outcome measures

The overhead motion places a large amount of stress on the shoulders of overhead athletes.^2^ To prevent shoulder joint distraction, the scapular and elbow muscles need to eccentrically contract to generate compressive forces.^30^ Failure of these muscles to sustain the repeated large magnitudes of eccentric contraction can lead to overuse injuries.^30^ This has fostered an interest in shoulder strength measures, with three studies investigating strength-based performance outcome measures^25^, ^26^, ^27^ and four studies were identified to have targeted strength as a training component in their IPP.^25^, ^26^, ^27^, ^28^

Of the seven significantly improved strength performance outcome measures, only three were observed in the dominant limb. The limited significant improvements to the dominant limb may be due to the sub-optimal training intervention period over which the IPPs were conducted. The IPPs that addressed strength measures were conducted over a duration of six (18 sessions)^26^^,^^27^ to eight weeks (16 sessions).^25^ A recent systematic review and meta-analysis of resistance training among youth athletes showed that strength improvement in youth athletes was more pronounced in programs with training periods of more than 23 weeks, with no significant differences between training frequencies of 1, 2, or 3 times per week.^31^ Therefore, future studies investigating strength-based performance outcome measures among youth athletes should consider a duration of 23 weeks or longer, with a frequency of at least once a week to enhance the methodological validity of strength measurements. Moreover, in comparison to the non-dominant limb, it is likely that the dominant limb of overhead athletes was already well-trained and consequently have lesser potential for muscular strength gain.^26^ Although reasonable, this hypothesis should be addressed in future studies by investigating within-subject differences in each limb to determine absolute strength gain.

The isokinetic strength testing speeds employed in the studies were different from the speeds at which the exercises in the program were performed. Based on the training principle of specificity,^25^ these differences could have impacted the results of the strength-based performance outcome measures and consequently led to non-significant findings. Additionally, despite performing the same action (e.g., concentric ER), different testing speeds were utilized for the isokinetic strength tests across studies (e.g., 60°/s and 240°/s), resulting in methodological differences. Regarding these differences, it may be purposeful to consider the use of isometric strength measures over the use of traditional gold-standard isokinetic strength measures. As the name suggests, isometric strength measures are measured isometrically, which removes the complexity surrounding testing speeds. This would lead to improved protocol consistency across studies, thus enabling researchers to draw valid conclusions while comparing the studies. Additionally, the preferred instrument for measuring isometric strength, the hand-held dynamometer (HHD), is low-cost and suitable for field use.^32^ This is in contrast to the equipment and procedures required in isokinetic strength measurements, which are expensive and cannot be used for on-field measurements.^32^ Utilising the HHD instead, therefore, enables practitioners such as coaches and trainers to use the device on-field as they may not always have access to specialised facilities and expert manpower. Moreover, the HHD has also demonstrated high intra- and inter-rater reliability for isometric shoulder strength measurements, making it a suitable alternative to an isokinetic device.^32^ This provides a reasonable basis for future studies interested in strength performance outcome measures to consider isometric strength measures and HHD over the traditional isokinetic strength measures.

### Mobility-based performance outcome measures

The interest in the range of motion (ROM) measurements stems from the importance of having adequate shoulder mobility for throwing performance, with greater shoulder mobility allowing a greater arc of motion through which the throwing arm can accelerate to produce high velocities at ball release or ball contact.^30^ However, as an adaptation to the repetitive overhead motion, the dominant shoulders of overhead athletes have been observed to demonstrate a decrease in IR ROM and an increase in ER ROM as compared to the non-dominant arm, while maintaining total ROM, in what is known as a ‘backward’ shift of the total arc of rotation.^33^ These adaptations are frequently discussed in the literature, specifically the development of glenohumeral internal rotation deficit (GIRD) and its association with injury.^34^, ^35^, ^36^ Interestingly, there is no real consensus to date, with a recent systematic review concluding only an association (non-significant correlation, *p* ​= ​0.06) between GIRD and upper extremity injury in overhead athletes.^37^ Despite this popularity of ROM measurements in overhead athletes, only two studies were identified to have investigated mobility-based performance outcome measures,^28^^,^^29^ while only one targeted mobility as a training component in their IPP.^29^

Thoracic kyphosis angle was one of the two mobility-based performance outcome measures that significantly improved compared to controls.^29^ This could be attributed to the dynamic thoracic mobility exercises (i.e., cat and dog exercise and trunk rotation exercise) performed in the mYKB-9 program. With the nature of overhead sports requiring repeated, coordinated use of the shoulder, it is essential for overhead athletes to improve thoracic kyphosis as a flexed thoracic spine results in a protracting scapula that alters shoulder mechanics.^38^ This would subsequently affect elbow mechanics due to the kinetic chain.^2^ It is recommended that future IPPs for overhead athletes include thoracic mobility exercises to reduce the risk of shoulder and elbow overuse injuries.

The second mobility-based performance outcome measure that significantly improved compared to controls was shoulder horizontal adduction (HA) ROM deficits.^29^ Theoretically, performing the posterior shoulder stretch (as part of the mYKB-9 program) should lead to reduced posterior shoulder tightness, and consequently improve the related problems of shoulder HA ROM deficits and GIRD.^33^ However, only the performance outcome measure of shoulder HA ROM deficits saw significant improvements, with no significant improvements for GIRD.^29^ This surprising outcome may be attributed to the way posterior shoulder tightness was targeted in mYKB-9. The cross-body stretch (also known as the HA stretch) and the sleeper stretch are generally recommended to reduce posterior shoulder tightness and improve HA and IR ROM^39^; however, neither of the stretches was included in the mYKB-9.^29^ This is in contrast to the original YKB-9 where the sleeper stretch was included, and yielded significant improvements in shoulder IR ROM deficits.^24^ The posterior shoulder stretch in the mYKB-9^29^ involved the athlete using their body weight to pull the shoulder into a stretch while being on all-fours (closed kinetic chain), which is different from the traditional cross-body stretch^39^ where the non-stretching arm pushes the stretching arm into HA while in a standing position (open kinetic chain). The difference in these stretching techniques could have affected the structure and ability of the posterior shoulder stretch to improve shoulder IR ROM. Recently, modifications to the traditional cross-body stretch and sleeper stretch have been suggested which involve stabilising the scapula to minimize the symptoms of pain and increasing the stretch.^39^ These modified stretches were effective at increasing shoulder HA and IR ROM among college baseball players.^40^ Future studies might benefit from including the modified sleeper stretch and cross-body stretch in their IPP to address shoulder HA and IR ROM deficits.

### Sport-specific performance outcome measures

Sport-specific performance outcome measures were investigated in four studies.^26^, ^27^, ^28^, ^29^ However, only two unique sport-specific performance outcome measures were assessed in the identified studies: serve accuracy and ball speed. Serve accuracy was explicitly evaluated in only one study, which reported non-significant improvements as compared to controls.^28^ This was also the only study to include plyometrics as a training component.^28^ However, during the measurements for ball speed in the other three studies, the athletes had to aim their serves or throws towards a target to attain accurate measures.^26^^,^^27^^,^^29^ Therefore, accuracy was indirectly incorporated during the measurements of ball speed. Nevertheless, explicitly assessing throwing or serving accuracy as a performance outcome measure would only serve to further enhance the specificity of the performance assessment of an athlete.

Significant improvements in ball speed as compared to controls were only observed in two studies.^28^^,^^29^ Interestingly, the mYKB-9 utilized in one of the studies did not include any strength-based or plyometric exercises, and only consisted of stretching, dynamic mobility, and lower extremity balance training exercises. It is likely that the focus on mobility as the sole training component in mYKB-9 contributed to addressing the ROM deficits and improved the kinetic chain of the overhead motion, resulting in improved ball speed.^38^ Future studies interested in improving sport-specific performance outcome measures of ball speed should consider mobility as a training component in their IPPs.

### Limitations

This systematic review is not without limitations. Language bias was present as only studies published in the English language were included. Due to differences in measurement protocols, a wide variety of performance outcome measures were found across the included studies. To address this limitation, similar performance outcome measures were categorised into a common category. For example, isokinetic concentric ER peak torque at 60°/s and isokinetic eccentric ER strength at 90°/s were both grouped into the category of strength performance outcome measures. This allowed for the evaluation of overall effectiveness of the IPPs on certain categories of performance outcome measures. However, the subjective classification of the performance outcome measures into categories may have introduced a bias. Similarly, the classification of exercises into training components was also subjective. A small number of eligible studies, variability in the number of exercises, repetitions, sets, frequency, and duration of intervention across the IPPs precluded the conduct of meta-analysis, which would have increased the strength of conclusions from this systematic review. Compliance with the prevention programs was not analyzed in this systematic review, which could have further contributed to understanding the effects of existing upper extremity IPPs on upper extremity performance outcome measures in overhead youth athletes.^6^^,^^41^

In employing detailed inclusion and exclusion criteria during study selection to provide the highest level of evidence, only five studies (covering a total of four sports) could be included in this systematic review concerning overhead youth athletes. Considering the existence of many other sports that feature the repetitive overhead motion, such as javelin, tchoukball, volleyball, and water polo, the small number of eligible studies and sports covered may be surprising. However, in a recent similar systematic review focusing on the effectiveness of shoulder IPPs among overhead athletes of all ages on shoulder injury risk, only seven studies (covering four sports) were found eligible.^42^ Therefore, the small number of studies eligible and sports covered in this systematic review may instead be an indication of the limited attention received by overhead sports in terms of the effectiveness of upper extremity IPPs on injury risk and performance outcome measures. Therefore, future research on injury prevention should consider focusing on overhead athletes and the prevention of overuse injuries to the upper extremity, particularly in overhead youth athletes who are at a greater risk of overuse injury.

This study only focused on training programs or exercises as the modality for injury prevention. With increasing attention given to training load as a modifiable risk factor for overuse injuries,^6^^,^^41^ future work on injury prevention efforts should investigate training load as an additional or alternative prevention modality, to understand its impact on reducing overuse injury risk.

The studies in this review consistently underperformed in Items 5, 6, and 7 of the PEDro scale, which is failure to blind all subjects, all therapists, and all assessors, respectively. While blinding all therapists (who administered the therapy) is not feasible due to the nature of the research, blinding the participants and assessors (who measured at least one key outcome) is possible. A suggestion would be to implement a sham exercise program for participants in the control group and ensure that the assessors and therapists involved in the study are unrelated.

## Conclusion

Existing upper extremity IPPs are effective at improving performance outcome measures of strength, mobility, and sports-specific measures. Strength-based performance outcome measures formed the majority of the outcome measures evaluated and had the highest overall effectiveness rate. The training components of the identified upper extremity IPPs were strength, mobility, and plyometrics, with strength being the most common training component. Overall, the studies included in this systematic review demonstrated adequate methodological quality and future upper extremity IPPs should include training components of strength, mobility, and plyometrics in their design given the effectiveness in significantly improving performance outcome measures of strength, mobility, and sport-specific measures. To allow comparisons across studies, standardized protocols should be established for the measurement and reporting of performance outcome measures, and the reporting of training components included in the IPP.

## Conflict of interests

The authors declare that they have no competing interests.

## Submission statement

Our work submitted has not been published previously, is not under consideration for publication elsewhere, its publication is approved by all authors and tacitly or explicitly by the responsible authorities where the work was carried out, and, if accepted, it will not be published elsewhere including electronically in the same form, in English or in any other language, without the written consent of the copyright holder.

## Authors’ contributions

RL contributed to the conception and design of the review. Both RL and SM conducted the analysis and interpretation of the data, the drafting and revising of the manuscript, agreed on the final draft, and take responsibility for the integrity and accuracy of the works.

## Ethical approval statement

Not applicable.

## Sources of funding

No sources of funding were used in the conduct of this study.
